# Effect of Tai Chi on sleep quality of cancer patients: a systematic review and meta-analysis

**DOI:** 10.3389/fneur.2026.1670047

**Published:** 2026-04-27

**Authors:** Yanjun Xu, Min Wang

**Affiliations:** Physical Education Department, Shanghai University of Finance & Economics, Shanghai, China

**Keywords:** cancer, meta-analysis, publication bias, sleep quality, Tai Chi

## Abstract

**Objective:**

The aim of meta-analysis was to analyze the impact of Tai Chi (TC) on the sleep quality of cancer patients.

**Methods:**

The randomized controlled trials (RCTs) reporting the effect of TC on sleep quality of cancer patients were searched from seven electronic databases, and their methodological quality was assessed. The sleep quality of cancer patients was evaluated using Pittsburgh sleep quality index (PSQI). Heterogeneity was tested by Cochran‘s Q and I^2^ test. Egger test assessed the publication bias. Sensitivity analysis was conducted to evaluate the effect of each study on the combined results.

**Results:**

Three RCTs with moderate methodological quality were included, which were executed in China and included a total of 145 patients. The pooled results showed that TC significantly improved the sleep quality of cancer patients [WMD (95%CI) = −3.34 (−4.42, −2.27), *p* < 0.0001]. No publication bias was observed among studies (*p* = 0.394).

**Conclusion:**

TC can improve the sleep quality of cancer patients. It is recommended to develop standardized protocols for TC and explore its long-term effects.

**Systematic review registration:**

https://www.crd.york.ac.uk/PROSPERO/view/CRD420251275343, identifier: CRD420251275343.

## Highlights

- Three RCTs were included in this meta-analysis.
- TC can improve the sleep quality of cancer patients.
- TC has potential application in clinical cancer treatment.

## Introduction

Cancer is a serious disease that can impact patients’ quality of life (QoL) across multiple areas, including their physical health, mental state, and social interactions. According to updated data from the International Agency for Research on Cancer (IARC), approximately 20 million new cancer cases were reported in 2022, along with 9.7 million cancer-related deaths (1). Improving the survival rate of cancer patients has always been a hot topic in medical research.

Cancer patients often accompany sleep disorders such as insomnia and sleep-related breathing disorder, which are caused by the cancer itself, panic mood, chemotherapy and surgery (2). These issues seriously decrease the QoL of patients. A prior meta-analysis indicated that 60.7% of participants (26,448 out of 46,279) experienced sleep disturbances, with a 95% confidence interval (95%CI) of 58.1–63.3%, underscoring the need for effective strategies to monitor and alleviate sleep issues in cancer patients to enhance their QoL (3). Furthermore, studies have shown that insomnia not only decreases the immunity of cancer patients but also promotes the expression of transforming growth factor beta (*TGF-β*), C-reactive protein (*CRP*), cyclooxygenase-2 (*COX-2*), and other cancer-related genes (4, 5). Insomnia is also recognized as an important prognostic factor for cancer (6). Therefore, seeking strategies to enhance the sleep quality of cancer patients is crucial for improving the overall effectiveness of cancer treatment.

At present, chemotherapy and exercise intervention are mainly used to prevent recurrence and metastasis of cancer after surgical treatment. However, chemotherapy can cause many adverse effects, such as nausea, vomiting, and peripheral neuropathy, imposing significant financial and psychological burdens on patients (7, 8). Conversely, physical activity is increasingly valued for its potential to improve cancer patients’ well-being, given its safety, accessibility, and various health benefits (9).

Tai Chi (TC) is a popular mind–body exercise based on the concept of “qi,” focusing on mood adjustment and relaxation of the spirit through gentle and smooth movements. This form of exercise is suitable for individuals of all health conditions globally, offering benefits for both health promotion and symptom management (10–12). Unlike traditional exercises that prioritize muscular strength and endurance, TC emphasizes mind–body integration through slow movements, full-body stretching, relaxation techniques, diaphragmatic breathing, and mental focus (13, 14). Recently, an increasing number of studies have focused on the impact of TC as a non-drug intervention for cancer patients (15, 16). TC can positively impact the quality of life and immune function of cancer patients (17, 18), as well as improve sleep quality (15, 19, 20). Although the results from these studies have indicated that TC may enhance the sleep quality of cancer patients, the small sample size and varying treatment protocols across these studies prevent drawing a definitive conclusion about its overall effectiveness.

Therefore, to reach a more objective conclusion, this study performed a meta-analysis by including a range high quality randomized controlled trials (RCTs) to evaluate the impact of TC on the sleep quality of cancer patients. The findings will offer scientific support for the potential use of TC in clinical cancer treatment.

## Methods

This meta-analysis was carried out based on the guidelines of the Preferred Reporting Items for Systematic Reviews and Meta-Analyses (PRISMA) statement. The protocol was registered on the PROSPERO platform with the code CRD420251275343.

### Literature search

The studies published before January 7, 2026 were systematically searched from PubMed, Embase, The Cochrane Library, Web of Science, Wanfang data, China National Knowledge Infrastructure (CNKI), and China Science and Technology Journal database (CQVIP) databases. Search terms included “neoplasms,” “cancer,” “sleep,” “Pittsburgh sleep quality index,” and “tai chi.” These terms words in the same category were combined with “OR,” while those in the different categories were combined with “AND.” Subject words were searched in combination with free words. Furthermore, to gather additional studies suitable for meta-analysis, references of related reviews and included literatures were screened. The search procedure for each electronic database is described in Supplementary Tables 1–5.

### Literature screening

*Inclusion criteria*: (1) cancer patients were diagnosed by a hospital, with no restrictions on cancer type and stage; (2) The intervention group performed TC; (3) The control group received treatment as usual (TAU); (4) The studies were RCTs; (5) Sleep quality was assessed as the primary outcome using the Pittsburgh sleep quality index (PSQI). PSQI consisted of 19 self-reported items across seven components about sleep quality. Each component is scored on a 0–3 scale, with a total score ranging from 0 to 21. The higher total score represents poorer overall sleep quality. Mean ± standard deviation was utilized to evaluate the difference in PSQI score after intervention. Alternatively, these values could be converted from other data reported in the literature.

*Exclusion criteria*: (1) Non-editorial studies such as reviews, conference abstracts, and editorials; (2) Studies that assessed sleep quality with other scales; (3) When multiple publications or data used in multiple articles, only the one with the most comprehensive data was included, and the others were excluded.

### Data extraction and quality assessment

According to the inclusion and exclusion criteria, the literature screening was independently completed by two investigators. Data extraction was executed according to the pre-designed standardized table after determining the included literature. The extracted information from each study included: study region, publication year, first author, sample size, the age and gender of participants, cancer treatment protocol, intervention time and frequency of TC, and outcome indicators. The extraction tables were exchanged to check by two investigators after both of them completed the data extraction. Any discrepancies were discussed and resolved.

The Cochrane Collaboration’s tool for assessing risk was used to assess the methodological quality of the included studies.

### Statistical analysis

The PSQI score was continuous data, and its combined effect was expressed as Weighted Mean Difference (WMD) and 95% confidence interval (CI). Given the high heterogeneity among the studies, a random-effects model was applied in meta-analysis.

Cochran’ s Q test and I^2^ test were used to evaluate the heterogeneity (21). If *p* < 0.05 and I^2^ > 50%, the heterogeneity was obvious among studies. Otherwise, the heterogeneity was not statistically significant.

The effects of cancer type, gender, and intervention duration on statistical heterogeneity and pooled effect size were evaluated using subgroup analysis.

The publication bias among studies was analyzed using Egger test (22). The method of one-by-one elimination in sensitivity analysis was used to evaluate whether each included study had a significant impact on the combined results.

The statistical analysis was performed by Review Manager (RevMan) (version 5.3, Copenhagen: The Nordic Cochrane Centre, The Cochrane Collaboation, 2020. available at revman.cochrane.org) and STATA software (version 17.0, StataCorporation, College Station, Texas, USA).

## Results

### Literature search

**Figure 1:**
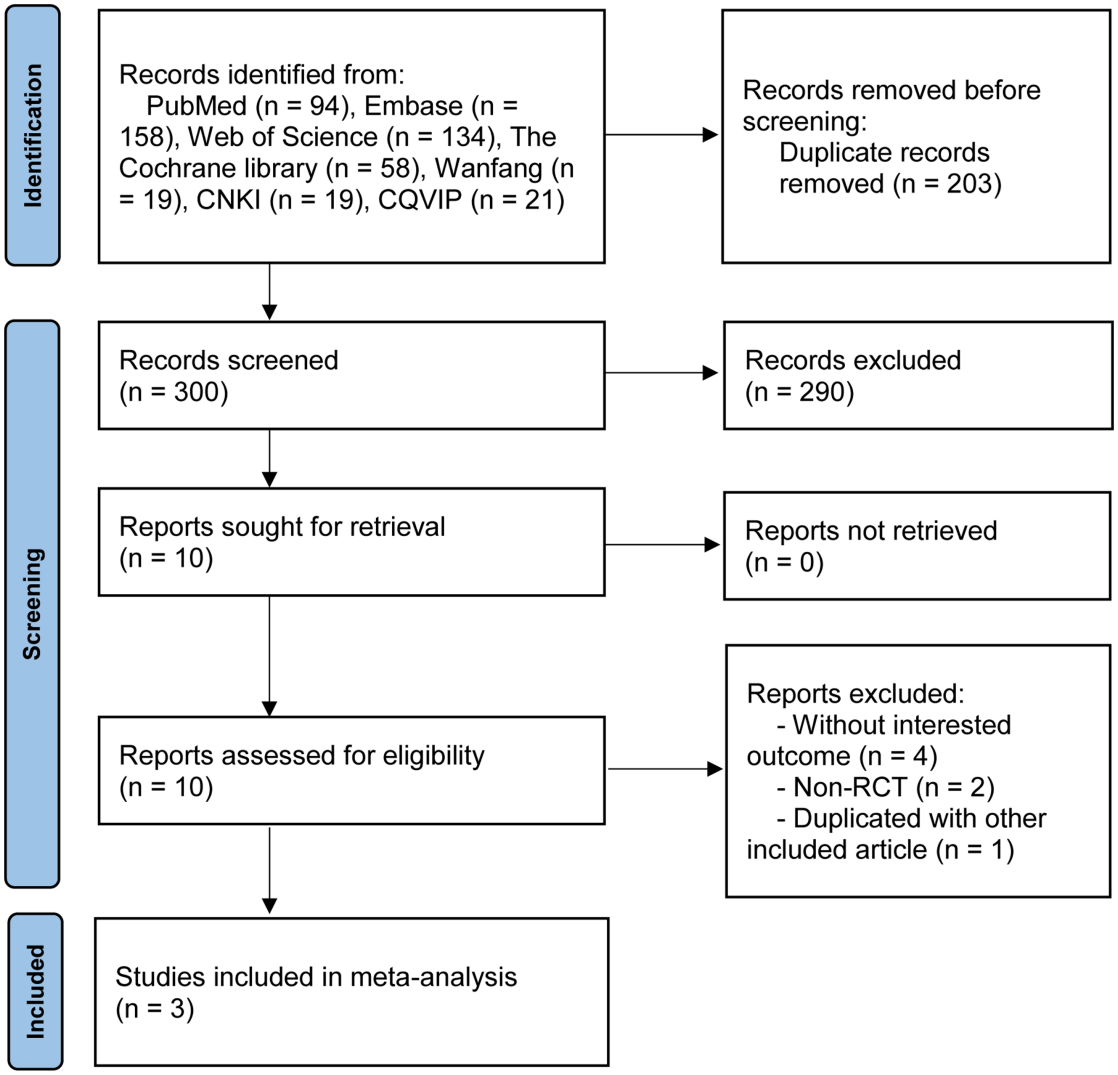
Flow chart of eligible articles selected for meta-analysis.

A total of 503 articles were screened out through the electronic database. There were 300 articles were reserved after eliminating 203 duplicate articles. After reviewing the title and abstract, 290 articles were excluded for not meeting the inclusion criteria. After further reading the full text, seven articles were excluded. Ultimately, three RCTs were included in this study (23–25). The process of literature search is shown in Figure 1.

### The characteristics of the included studies

The included studies were all executed in China, with sample sizes ranging from 20 to 72 cases and a total of 145 patients. Of these, 71 were assigned to the intervention group and 74 to the control group. Age and gender were comparable between the two groups. The specific cancer types, treatment protocols, and intervention programs of TC are detailed in Table 1.

**Table 1:** Characteristics of the 3 included studies.

Study	Country	Type of cancer	Treatment	Duration	Group	Intervention	*n*, M/F	Age, years
Cheng et al., 2021 (24)	China	Lung, gastric, breast cancer	2–4 courses of chemotherapy and/or radiation therapy	12 weeks	TC	The 24-form Yang-style TC (30 min), > = 3 days/week	26, 16/10	64.7 ± 7.6
					Control	As usual	27, 13/14	67.8 ± 7.3
Cheung et al., 2021 (25)	China	Advanced lung cancer	Targeted therapy, Chemotherapy or Radiotherapy	12 weeks	TC	The 24-form Yang-style TC (60 min), twice/week	9, 6/3	61.11 ± 7.01
					Control	As usual	11, 5/6	58.36 ± 9.32
Yao et al., 2022 (23)	China	Breast cancer	Adjuvant chemotherapy	8 weeks	TC	The 8-form Yang Style TC (60 min), 2 days/week	36, 0/36	45.3 ± 8.5
					Control	As usual	36, 0/36	48.6 ± 7.8

TC, Tai Chi; M, male; F, female.

The quality evaluation results are displayed in Supplementary Figures 1A,B. One study (25) did not report the specific allocation concealment schemes. Due to the difficulty of implementation, all included studies (23–25) did not employ blinding for researchers and subjects. One study (24) did not implement blinding for outcome assessments. Therefore, there was a moderate bias in selection, performances, and detection. Overall, the bias risk of included studies was moderate.

### Results of meta-analysis

**Figure 2:**
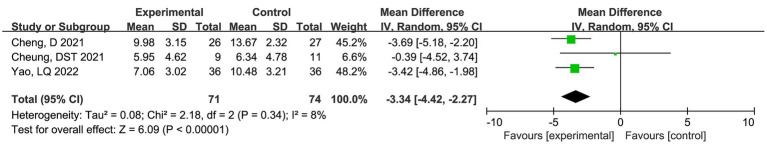
Forest plot showing the effect of the TC intervention on sleep quality of cancer patients.

The PSQI scores showed a decrease in the TC group in relative to the control group after intervention [WMD (95%CI) = −3.34 (−4.42, −2.27), *p* < 0.0001] (Figure 2), indicating that TC could improve the sleep quality of cancer patients. No significant heterogeneity existed among the included studies (I^2^ = 8%, *p* = 0.34).

### Subgroup analysis

**Figure 3:**
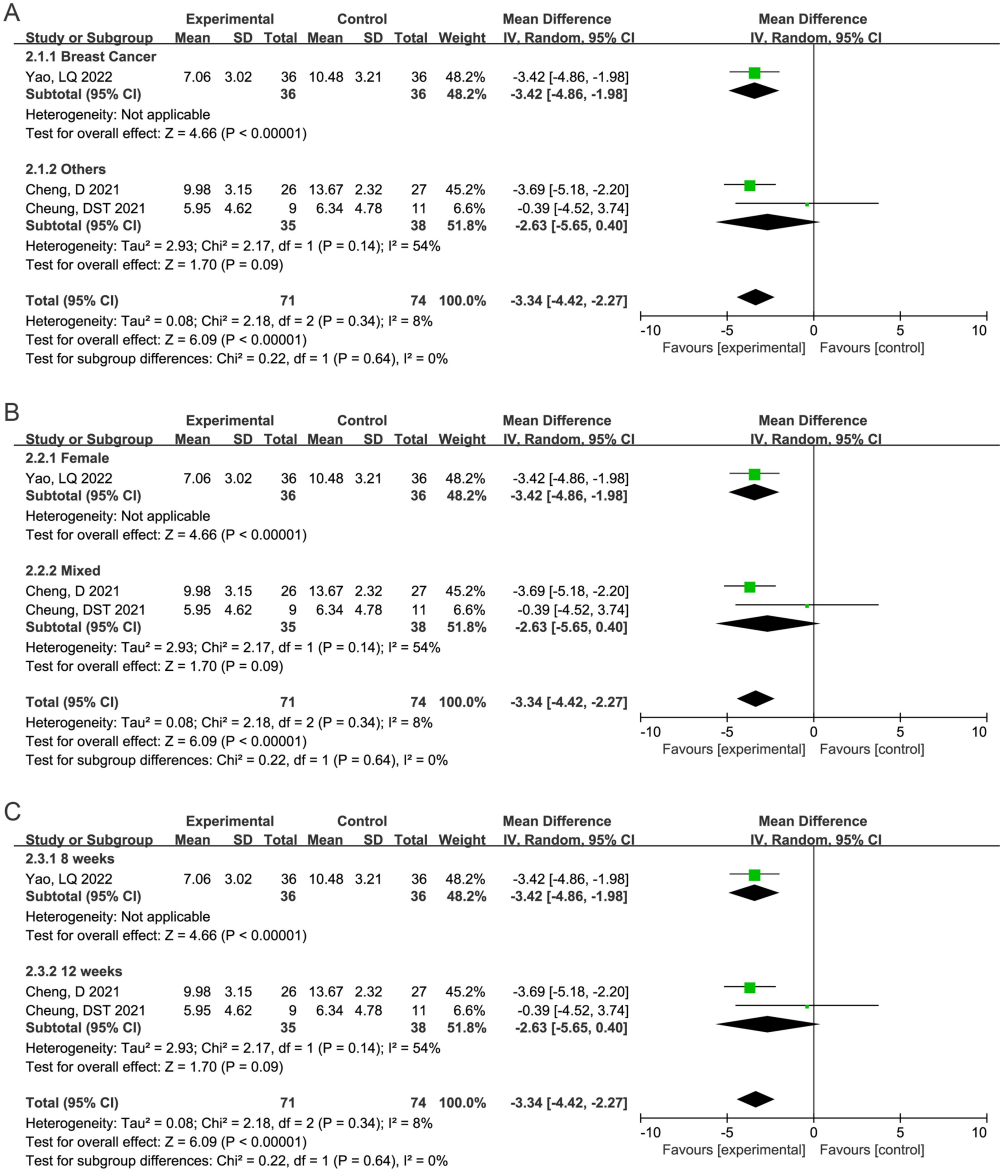
Subgroup analysis based on cancer types **(A)**, gender **(B)**, and intervention duration **(C)**.

Sensitivity analysis detected the source of heterogeneity. Figures 3A–C showed the results of subgroup analysis based on cancer types, gender, and intervention duration. In the study by Cheng et al. (25), although breast cancer patients were included, their PSQI total score was not reported. As these patients represented a small proportion of the overall sample, they were categorized as “Others” in the cancer types subgroup analysis. Due to the inclusion of only 3 studies, the subgroup composition of the three variables was completely consistent. In female breast cancer patients, after 8 weeks of TC, the total PSQI score was significantly lower than that of the control group [WMD (95% CI) = −3.42 (−4.86, −1.98), *p* < 0.0001]. In the remaining 2 studies, which included patients with various types of cancer, both male and female participants engaged in 12 weeks of TC, and there was no significant difference in the total PSQI score compared to the control group [WMD (95% CI) = −2.63 (−5.65, 0.40), *p* = 0.09].

### Publication bias test and sensitivity analysis

**Figure 4:**
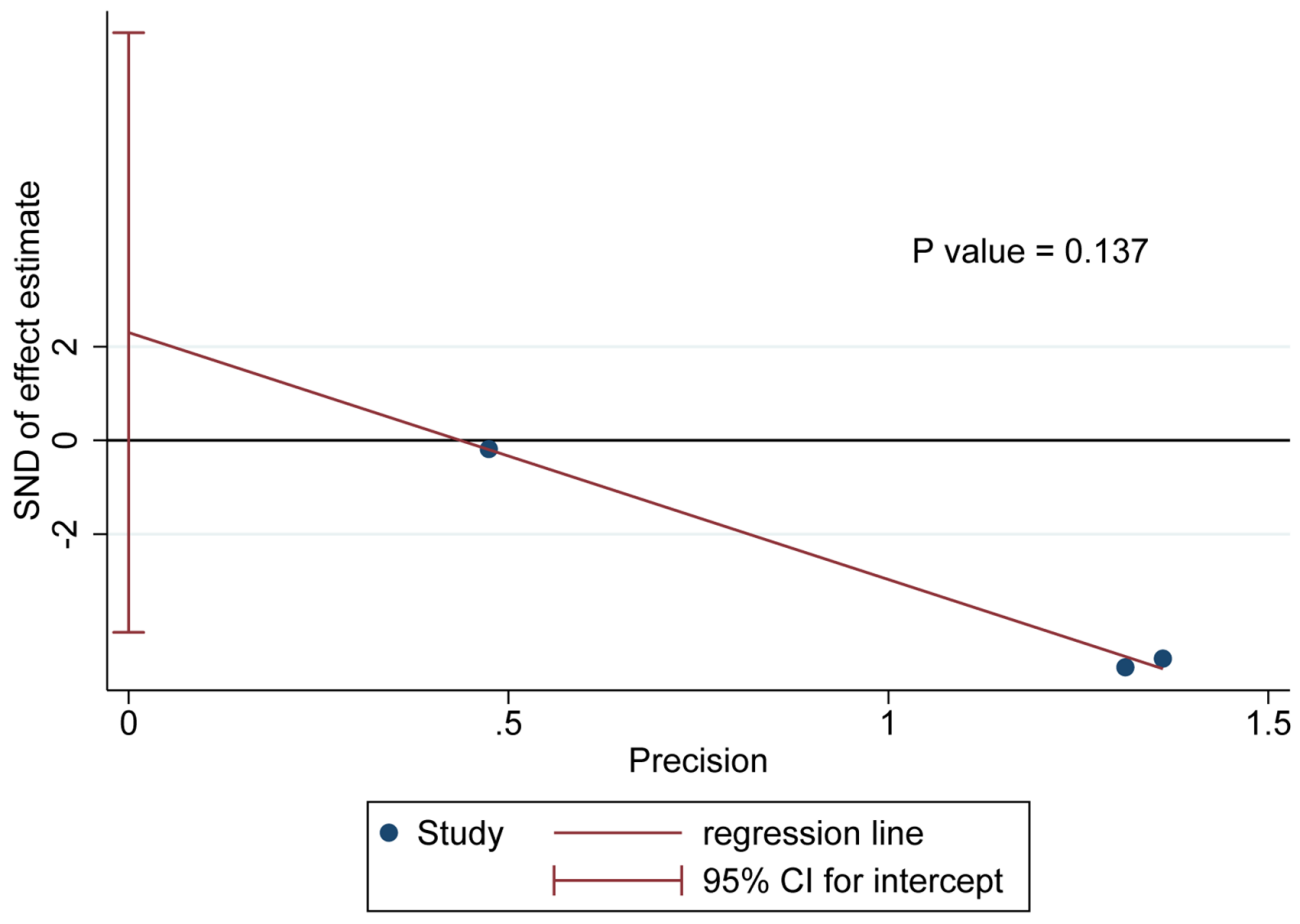
Publication bias evaluation for included studies.

**Figure 5:**
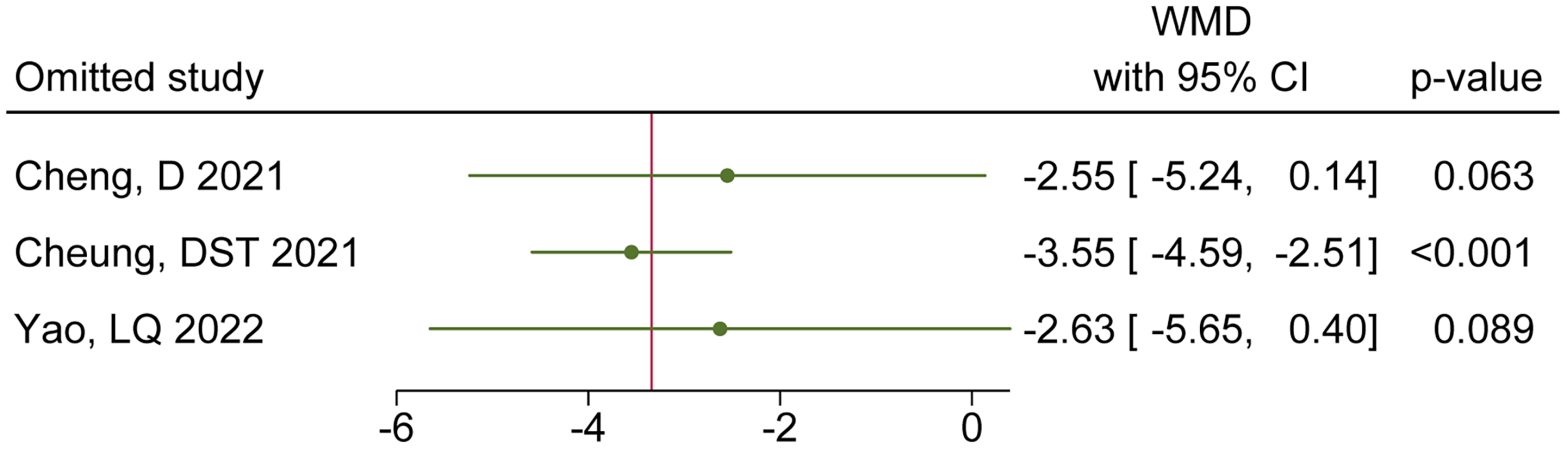
Sensitivity analysis showing the effect of each study on the pooled results.

The Egger test results for the outcome indicator indicated no significant publication bias (*p* = 0.137, Figure 4). The result of sensitivity analysis showed that though the one-by-one exclusion method, the range of variation in the combined result was as follows: WMD (95%CI) = −3.55 (−4.59, −2.51) to −2.55 (−5.24, 0.14). After excluding Cheng et al. or Yao et al., the combined results of the remaining studies reversed to be not statistically significant, indicating poor robustness of the meta-analysis results (Figure 5).

## Discussion

Sleep disturbances and related psychological symptoms, which negatively impact survival, are chronic issues for cancer patients (26, 27). With the evolution of modern medical model and treatments, exercise intervention has emerged as a crucial component of cancer care, including sleep disturbances, which can avoid potential adverse effects from pharmacological treatments (28, 29). Proper exercise can promote blood circulation, accelerate body metabolism, strengthen the immune system, and alleviate anxiety among patients (30). TC is a popular mind–body exercise that can promote physical and psychological well-being (31, 32). However, there is a scarcity of studies assessing the impact of TC on sleep quality of cancer patients. In this meta-analysis, we found that TC exhibited a positive effect on the sleep quality of cancer patients; however, more large-scale RCTs were needed to be conducted in other regions to validate the generalizability of the results.

TC is a form of aerobic exercise that focuses on mindfulness, controlled breathing, and slow movements of the lambs (33). As a form of health promotion, TC has garnered significant interest in the realm of Chinese traditional spots culture and medical rehabilitation, particularly in the context of cancer (34). Research has demonstrated that TC can effectively support cancer patients in their post-surgery recovery and alleviate cancer-related fatigue (35). Research suggests that TC can enhance the sleep quality of cancer patients in several ways. (1) TC may stimulate the brain to produce catecholamines, which can alleviate tension in the nervous system and decrease stress level (20). Long-term TC exercise has been linked to increased dopamine secretion from substantia nigra cell (36), which helps alleviate negative emotions like depression and anxiety, ultimately improving patients’ sleep quality (37); (2) TC can decrease the number of pro-inflammatory monocytes while increasing the count of regulatory T cells, resulting in a reduction inflammatory responses and an enhancement of sleep quality (36). We found that TC effectively improved the sleep quality of cancer patients, offering a scientific foundation for integrating TC into cancer treatment protocols.

Researches have indicated that breast cancer patients undergoing radical operations involving breast and myofascial removal, as well as axillary lymph node dissection, may suffer significant damage to the lymphatic network, leading to lymphedema on the affected side (38). TC has been found to enhance T lymphocyte proliferation, boost immune function, and consequently improve sleep quality in these patients (36). Moreover, engaging in appropriate aerobic exercise can aid in wound healing. Research also indicates that post-surgery, individuals with breast cancer often experience low self-esteem, anxiety, and depression, all of which can negatively impact recovery and quality of life (39). TC has the potential to regulate emotions and help maintain a positive mental state in patients (37). Additionally, cancer-specific exercise guidelines recommend that cancer patients engage in aerobic exercise at least three times a week for 30 min each session, at a moderate intensity, for a duration of 8–12 weeks (40). However, adjustments should be made based on the cancer stage and patient’s physical condition. Li-Qun Yao et al. (24) indicated that a TC group could significantly improve the sleep quality of patients with 60-min sessions twice a week for 8 weeks. Mc Quade et al. (41) also observed improved sleep quality in patients with TC + Qigong interventions over the short term after 8 weeks. Regrettably, our results showed poor robustness of the meta-analysis results for these factors. Therefore, we will continue to collect more comprehensive data and further explore the association between the sleep quality of cancer patients and the TC intervention in treatment duration and different cancer types.

This study has certain strengths. Firstly, only RCTs were included, and the methodological quality of the studies was high. Secondly, this study used a standardized sleep quality assessment tool and employed WMD as the effect size, and the differences in the intervention effects between the TC group and the control group could be clearly delineated. However, there were several limitations, as follows: (1) The number of included studies were small and these studies were all from China. It is necessary to validate the generalizability of the meta-analysis results in other countries; (2) Although subgroup analyses were conducted, the small number of studies made it difficult to accurately assess the impact of cancer type, gender, and intervention duration on the intervention effects; (3) The study did not assess the long-term benefits of TC on sleep quality in cancer patients due to insufficient data; (4) The included studies showed differences in the TC type of Tai Chi, exercise duration, intervention frequency, and intervention period. It is recommended to explore and establish a standardized TC intervention protocol in future studies; (5) The study established the PSQI as the sole assessment tool because it was the most commonly utilized tool for assessing sleep quality among the studies identified during our preliminary literature search. The detailed literature screening revealed no studies that assessed sleep quality in cancer patients undergoing Tai Chi using Polysomnography (PSG) or actigraphy, with only 1–2 studies employing alternative tools, such as the Medical Outcomes Study Sleep Scale. To minimize heterogeneity among the included studies and ensure the interpretability of the meta-analysis results, only studies using the PSQI were included, which may limit the physiological depth of the findings. Future research should explore objective measures of sleep quality.

## Conclusion

In conclusion, TC could enhance the sleep quality of cancer patients. More high-quality, larger-sample trials should be performed to confirm the results and potential long-term effects of TC on sleep quality in cancer patients.

## Data Availability

The original contributions presented in the study are included in the article/Supplementary material, further inquiries can be directed to the corresponding author.
