# Supplementary figures and images for: Effect of Tai Chi on sleep quality of cancer patients: a systematic review and meta-analysis
Source: Front Neurol. 2026 Apr 27;17:1670047. doi: 10.3389/fneur.2026.1670047 (PMC13159528; doi:10.3389/fneur.2026.1670047)

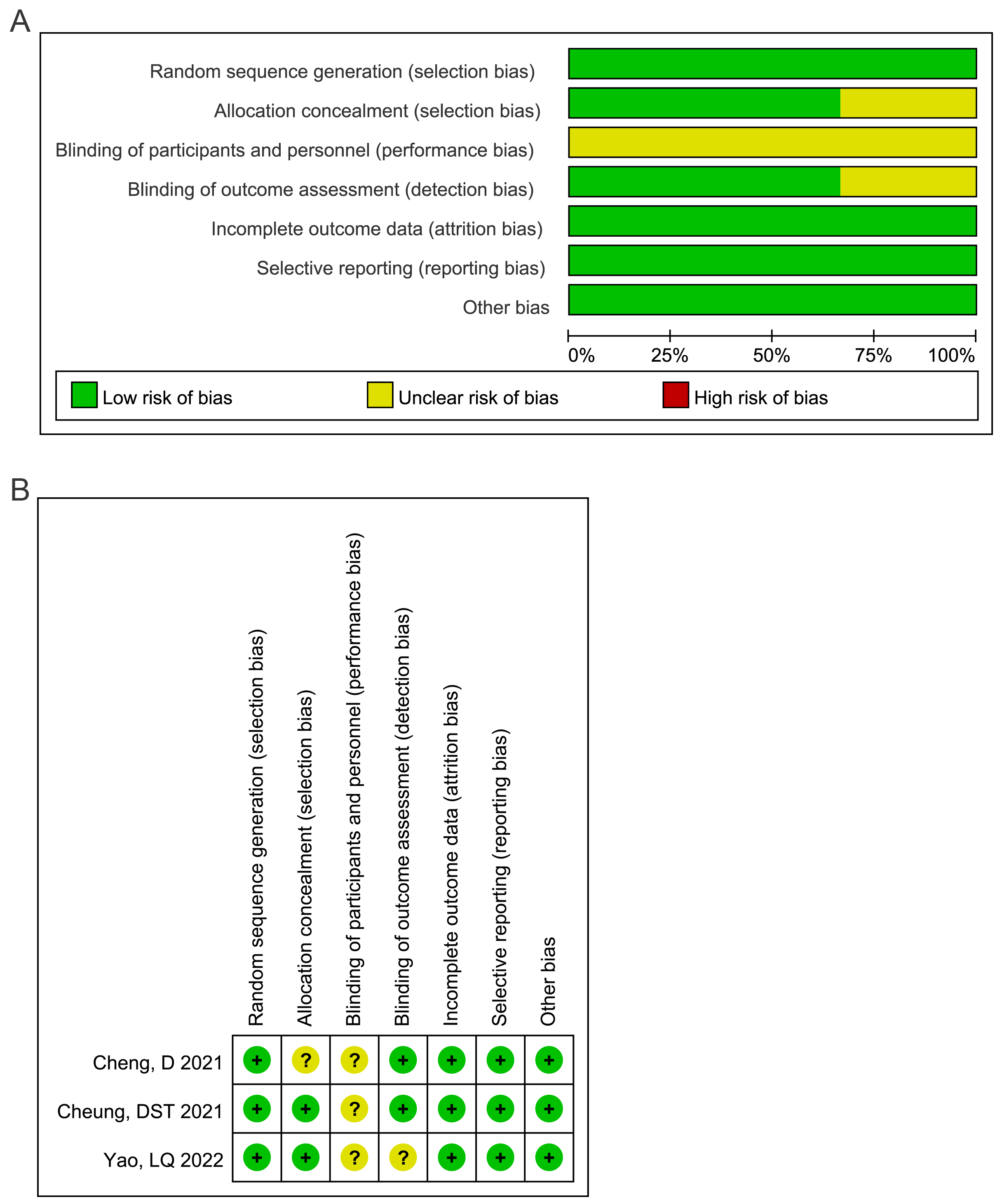

Supplement: Supplementary file 1 [file Image_1.tif]
