# Supplementary material for: Effect of Tai Chi on sleep quality of cancer patients: a systematic review and meta-analysis
Source: Front Neurol. 2026 Apr 27;17:1670047. doi: 10.3389/fneur.2026.1670047 (PMC13159528; doi:10.3389/fneur.2026.1670047)
Supplement: Supplementary file 2 [file Table_1.docx]

**Supplementary Table 1 Search strategy and results of PubMed** **(the retrieval time: 20260107)**

| Search | Query | Items found |
| --- | --- | --- |
| #1 | "tai ji"[MeSH Terms] OR ("tai"[All Fields] AND "ji"[All Fields]) OR "tai ji"[All Fields] OR ("tai"[All Fields] AND "chi"[All Fields]) OR "tai chi"[All Fields] | 5233 |
| #2 | "neoplasms"[MeSH Terms] OR "neoplasms"[All Fields] OR "neoplasm"[All Fields] OR "cancer"[All Fields] OR "cancers"[All Fields] OR "cysts"[MeSH Terms] OR "cysts"[All Fields] OR "cyst"[All Fields] OR "tumour"[All Fields] OR "tumor"[All Fields] OR "tumours"[All Fields] OR "tumors"[All Fields] | 6188883 |
| #3 | "sleep"[MeSH Terms] OR "sleep"[All Fields] OR "PSQI"[All Fields] OR "Pittsburgh sleep quality index"[All Fields] | 333261 |
| #4 | #1 AND #2 AND #3 | 94 |

**Supplementary Table 2 Search strategy and results of Embase (the retrieval time: 20260107)**

| Search | Query | Items found |
| --- | --- | --- |
| #1 | ('tai ji'/exp OR 'tai ji' OR 'tai chi'/exp OR 'tai chi') | 4766 |
| #2 | ('cancer'/exp OR cancer OR 'tumour'/exp OR tumour OR 'tumor'/exp OR tumor OR 'neoplasm'/exp OR neoplasm OR 'cyst'/exp OR cyst) | 7657907 |
| #3 | ('sleep'/exp OR sleep OR 'psqi'/exp OR psqi OR 'pittsburgh sleep quality index'/exp OR 'pittsburgh sleep quality index') | 543796 |
| #4 | #1 AND #2 AND #3 | 158 |

**Supplementary Table 3 Search strategy and results of Web of Science (1985-present) (the retrieval time: 20260107)**

| Search | Query | Items found |
| --- | --- | --- |
| #1 | "tai ji" OR "tai chi"(All Fields) | 5723 |
| #2 | "neoplasms" OR "neoplasm" OR "cancer" OR "cancers" OR "cysts" OR "cyst" OR "tumour" OR "tumor" OR "tumours" OR "tumors" (All Fields) | 4830652 |
| #3 | sleep OR PSQI OR "Pittsburgh sleep quality index" (All Fields) | 395354 |
| #4 | #1 AND #2 AND #3 | 134 |

**Supplementary Table 4 Search strategy and results of The Cochrane library (the retrieval time: 20260107)**

| Search | Query | Items found |
| --- | --- | --- |
| #1 | MeSH descriptor: [Tai Ji] explode all trees | 638 |
| #2 | (tai ji OR tai chi):ti,ab,kw (Word variations have been searched) | 2194 |
| #3 | #1 OR #2 | 2194 |
| #4 | MeSH descriptor: [Neoplasms] explode all trees | 131246 |
| #5 | MeSH descriptor: [Cysts] explode all trees | 3285 |
| #6 | (cancer OR tumour OR tumor OR neoplasm OR cyst OR cancers OR tumours OR tumors OR neoplasms OR cysts):ti,ab,kw (Word variations have been searched) | 288190 |
| #7 | #4 OR #5 OR #6 | 302204 |
| #8 | MeSH descriptor: [Sleep] explode all trees | 9392 |
| #9 | (sleep OR PSQI OR "Pittsburgh sleep quality index"):ti,ab,kw (Word variations have been searched) | 64958 |
| #10 | #8 OR #9 | 65126 |
| #11 | #3 AND #7 AND #10 | 59 |
| #12 | #11 in Trials | 58 |

**Supplementary Table 5 Search strategy and results of Chinese databases (the retrieval time: 20260107)**

| Database | Query | Items found |
| --- | --- | --- |
| WANFANG | Terms: (Tai Ji) AND (Title or Keywords:(cancer) OR Title or Keywords:(tumour)) AND (Terms:(sleep) OR (All:(PSQI)) | 19 |
| CNKI | (SU%= Tai Ji OR TI%= Tai Ji OR TI%= Tai Ji Quan) AND (TKA%=tumour OR TKA%=cancer OR TI%=carcinoma) AND (TKA%=sleep OR FT%=PSQI) | 19 |
| CQVIP | (U=Tai Ji OR U= Tai Ji Quan) AND (U=tumour OR U=cancer OR U= carcinoma) AND (U=sleep OR U=PSQI) | 21 |
